# Comparison of methods for the detection of biofilm production in coagulase-negative staphylococci

**DOI:** 10.1186/1756-0500-3-260

**Published:** 2010-10-14

**Authors:** Adilson Oliveira, Maria de Lourdes RS Cunha

**Affiliations:** 1 UNESP - Univ Estadual Paulista, Department of Microbiology and Immunology, Biosciences Institute Bacteriology Laboratory, Botucatu, SP, Brazil

## Abstract

**Background:**

The ability of biofilm formation seems to play an essential role in the virulence of coagulase-negative staphylococci (CNS). The most clearly characterized component of staphylococcal biofilms is the polysaccharide intercellular adhesin (PIA) encoded by the *icaADBC *operon. Biofilm production was studied in 80 coagulase-negative staphylococci (CNS) strains isolated from clinical specimens of newborns with infection hospitalized at the Neonatal Unit of the University Hospital, Faculty of Medicine of Botucatu, and in 20 isolates obtained from the nares of healthy individuals without signs of infection. The objective was to compare three phenotypic methods with the detection of the *icaA*, *icaD *and *icaC *genes by PCR.

**Findings:**

Among the 100 CNS isolates studied, 82% tested positive by PCR, 82% by the tube test, 81% by the TCP assay, and 73% by the CRA method. Using PCR as a reference, the tube test showed the best correlation with detection of the *ica *genes, presenting high sensitivity and specificity.

**Conclusions:**

The tube adherence test can be indicated for the routine detection of biofilm production in CNS because of its easy application and low cost and because it guarantees reliable results with excellent sensitivity and specificity.

## Background

Coagulase-negative staphylococci (CNS) are the microorganisms most frequently involved in nosocomial infections among neonates. These infections are generally associated with the use of catheters and other medical devices [1]. The capacity to adhere to polymer surfaces and consequent biofilm production are the main virulence factors of CNS, especially *S. epidermidis*, the most frequently isolated species.

The biofilm protects CNS against the action of antibiotics administered for the treatment of these infections and also against the patient's immune system. Removal of the foreign body is often necessary for cure [2,3]. In this respect, CNS infections seem to be related to the health condition of the patient and to the production of this extracellular polysaccharide [4,5].

The biofilm consists of layers of cell clusters embedded in a matrix of extracellular polysaccharide, called polysaccharide intercellular adhesin (PIA), which consists of β-1,6-N-acetylglycosamine and is synthesized by N-acetylglucosaminyltransferase [6]. PIA is involved in cell-cell adhesion and is essential for biofilm production by CNS, which is observed in most clinical strains of *S. epidermidis *[7,8].

The synthesis of PIA is mediated by the products of the chromosomal *ica *gene (intercellular adhesion), which are organized in an operon structure. This operon contains the *icaADBC *genes, in addition to the *icaR *gene which exerts a regulatory function and is transcribed in the opposite direction. Once this operon is activated, four proteins are transcribed, IcaA, IcaD, IcaB and IcaC, which are necessary for the synthesis of PIA [9-11]. PIA is synthesized from UDP-N-acetylglucosamine by N-acetylglucosaminyltransferase, which is encoded by the *ica *locus, particularly *icaA*. The expression of this gene alone induces low enzymatic activity and the production of low amounts of polysaccharide. However, the simultaneous expression of *icaA *and *icaD *promotes a significant increase in N-acetylglucosaminyltransferase, with a consequent increase in the amount of polysaccharide, forming oligomers of 10-20 β-1,6-N-acetylglucosamine residues [12,13]. The *icaC *gene, when expressed concomitantly with *icaA *and *icaD*, induces the synthesis of longer oligomers that contain up to 130 residues. The *icaB *gene probably encodes a secretory protein that does not seem to be involved in the biosynthesis of PIA [14,15].

Recent studies have shown that additional components, such as accumulation-associated protein (Aap), DNA and RNA independently or in cooperation with the *ica *operon, have also been suggested to be important in CNS biofilms [16-18]. Bap [biofilm-associated protein] has been shown to be involved in the initial attachment, intercellular adhesion and biofilm formation of *S. aureus *[19]. Interestingly, Bap homologue protein (Bhp) is found in human *S. epidermidis *strains and induces an alternative mechanism of biofilm formation that does not depend on PIA [20].

Various methods are currently used in medical areas for the detection of biofilm production, including visual assessment by electron microscopy using different types of microscopes. The most versatile and effective nondestructive approach for studying biofilms is confocal laser scanning microscopy (CLSM). CLSM markedly reduces the need for pretreatments such as disruption and fixation, which reduce or eliminate the evidence of microbial relationships, complex structures and biofilm organization, without the limitations encountered with scanning electron microscopes [21-23]. Bridier *et al. *[24] proposed CLSM combined with the use of 96-well microtiter plates compatible with high resolution imaging for the study of biofilm formation and structure. The authors reported that the combined use of microplates and confocal imaging proved to be a good alternative to other high throughput methods commonly used since it permits the direct, *in situ *qualitative and quantitative characterization of biofilm architecture.

However, qualitative methods, such as the tube adherence test described by Christensen *et al. *[25] and the Congo red agar (CRA) method described by Freeman *et al. *[26], and quantitative methods such as the tissue culture plate (TCP) assay described by Christensen *et al. *[27] are used in routine laboratories. Molecular techniques such as the polymerase chain reaction (PCR), which amplifies the genes involved in biofilm production, complement these methods.

The objective of the present study was to investigate biofilm production in CNS strains isolated from clinical specimens of newborns hospitalized at the Neonatal Unit of the University Hospital, Faculty of Medicine of Botucatu, and in isolates obtained from the nares of healthy individuals by three phenotypic methods and by PCR for detection of the *icaA*, *icaD *and *icaC *genes.

## Materials and methods

### Strains

A total of 100 CNS isolates were studied, including 80 isolated from clinical specimens obtained from newborns hospitalized at the Neonatal Unit of the University Hospital, Faculty of Medicine, Universidade Estadual Paulista (UNESP), Botucatu Campus, and 20 obtained from the nares of healthy subjects. The following international reference strains were used as controls: the non-biofilm producers *S. epidermidis *ATCC 12228 and *S. warneri *ATCC 10209 (negative control), and the biofilm producers *S. epidermidis *ATCC 35983, *S. simulans *ATCC 27851 and *S. xylosus *ATCC 29979 (positive control).

### Identification of coagulase-negative staphylococci

The isolates obtained from the clinical specimens were seeded onto blood agar and stained by the Gram method for the determination of purity, morphology and specific staining. After confirmation of these characteristics, the strains were submitted to catalase and coagulase tests. The genus *Staphylococcus *was differentiated from *Micrococcus *according to the method described by Baker *et al. *[28].

The simplified scheme proposed by Cunha *et al. *[29] was used for the identification of CNS based on sugar utilization tests. After species confirmation, the isolates were stored in nutrient broth with glycerol in a freezer at -70°C.

### Study of biofilm production

#### Investigation of biofilm production by adherence to borosilicate test tubes (Christensen *et al. *1982)

Biofilm production was investigated by the tube adherence test proposed by Christensen *et al. *[25]. A positive result was defined as the presence of a layer of stained material adhered to the inner wall of the tubes. The exclusive observation of a stained ring at the liquid-air interface was not considered to be positive.

#### Investigation of biofilm production by adherence to polystyrene plates (modified method of Christensen *et al. *1985)

The quantitative method of adherence to polystyrene plates (TCP) proposed by Christensen *et al. *[27] was also used in the present study, with modifications. These modifications included an increase in the glucose concentration of TSB from 0.25% to 2%, a longer incubation period (24 h instead of 18 h), and determination of optical density in dry plates and plates washed with 95% ethanol using filters of 492 and 540 nm. Analysis of the sensitivity and specificity of the TCP method using PCR as a parameter showed that the use of dry plates and of the 540-nm filter provided the best sensitivity (97.6%) and specificity (94.4%) when compared to ethanol-containing plates and the 492-nm filter, and was therefore chosen for analysis of the results.

The isolates were classified into three categories: non-adherent, optical density equal to or lower than 0.111; weakly adherent, optical density higher than 0.111 or equal to or lower than 0.222; strongly adherent, optical density higher than 0.222.

When the cut-off corresponded to non-adherent the isolates were classified as negative, and as positive when the cut-off corresponded to weakly or strongly adherent. Sensitivity and specificity were calculated for each situation in relation to the concomitant presence of the *icaA *and *icaD *or *icaADC *genes using PCR as the reference method.

#### Investigation of biofilm production by the Congo red agar method proposed by Freeman *et al. *(1989)

Phenotypic characterization of biofilm production was performed by culture of the CNS isolates on CRA plates as proposed by Freeman *et al. *[26]. According to the authors, biofilm producers form black colonies on CRA, whereas non-producers form red colonies. The Congo red dye directly interacts with certain polysaccharides, forming colored complexes [30].

A five-color reference scale was used to accurately determine all color variations shown by the colonies. Isolates presenting two tones of black, bright black (BB) and dry-opaque black (OB), were classified as biofilm producers, whereas red, pink and bordeaux colonies were classified as negative. In some cases, red and bordeaux subcolonies arose in the center of black colonies (BB) after 48 h of culture. These colonies were removed and subcultured for 24 h on CRA to obtain pure isolates of the producer and non-producer variants. These isolates were also submitted to the phenotypic tests (tube adherence test and TCP assay) and to detection of the *ica *genes by PCR.

#### Detection of the *icaA*, *icaC *and *icaD *genes specific for biofilm production

The procedure used for detection of the *ica *genes involved the following steps: nucleic acid extraction using the Illustra kit (GE Healthcare), PCR amplification according to the parameters described by Arciola *et al. *[31], and visualization of the amplified products by gel electrophoresis.

### Statistical analysis

Sensitivity and specificity [32] were evaluated by comparing the phenotypic methods used for the detection of biofilm production and PCR for the detection of the genes involved in biofilm synthesis. All isolates carrying at least two of the genes studied were considered to be positive for biofilm production. Sensitivity and specificity were evaluated as follows: sensitivity - proportion of PCR-positive isolates (concomitant presence of the *icaA *and *icaD *or *icaADC *genes) that tested positive by the other phenotypic methods; specificity - proportion of PCR-negative isolates (no detection of the *ica *gene) that tested negative by the phenotypic methods.

Agreement between the tests and the presence of the *ica *genes was evaluated using the kappa index [33,34].

## Results

### Isolates

A total of 100 CNS isolates were studied, including 80 isolated from clinical specimens obtained from newborns hospitalized at the Neonatal Unit of the University Hospital, Faculty of Medicine, Universidade Estadual Paulista (UNESP), Botucatu, Brazil, and 20 obtained from the nares of healthy subjects. Among the 80 isolates obtained from clinical specimens, 50 were isolated from catheter tips and 30 by blood culture.

### Identification of coagulase-negative staphylococci

*S. epidermidis *was the most frequently detected species (81%), followed by *S. cohnii *(4%), *S. saprophyticus *(4%), *S. warneri *(4%), *S. haemolyticus *(2%), *S. xylosus *(2%), *S. capitis *(2%), and *S. lugdunensis *(1%).

Table 1 shows the frequency of CNS species according to clinical specimen. *S*. *epidermidis *was the CNS species most frequently isolated from clinical specimens (catheter and blood culture) and from nares specimens of healthy subjects. *S. haemolyticus*, *S. xylosus*, *S. lugdunensis *and *S. capitis *were also isolated from clinical specimens, whereas *S. cohnii *and *S. saprophyticus *were only isolated from the nares of healthy subjects. *S*. *warneri *was isolated from blood culture and nares specimens.

**Table 1:** Frequency of coagulase-negative staphylococci isolated from different clinical specimens

Species	Catheter tip	Blood culture	Nasal fossa	Overall frequency	Percentage (%)
*S. epidermidis*	45	27	9	81	81
*S. cohnii*	0	0	4	4	4
*S. haemolyticus*	1	1	0	2	2
*S. saprophyticus*	0	0	4	4	4
*S. warneri*	0	1	3	4	4
*S. xylosus*	2	0	0	2	2
*S. lugdunensis*	0	1	0	1	1
*S. capitis*	2	0	0	2	2

**Total**	50	30	20	100	100

### Study of biofilm production

#### Detection of the *icaA*, *icaC *and *icaD *genes specific for biofilm production

**Figure 1:**
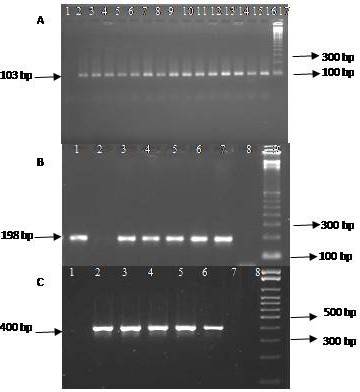
**Agarose gel electrophoresis of PCR products stained with Syber Safe **: A → *icaA *gene (103 bp), 1: negative control, 2-15: positive samples, 16: positive control and 17: molecular weight (100 bp), B → *icaD *gene (198 bp), 1: positive sample, 2: negative sample, 3-6: positive samples, 7: positive control, 8: negative control and 9: molecular weight (100 bp), C → *icaC *gene (400 bp), 1: negative sample, 2-5: positive samples, 6: positive control, 7: negative sample, 8: negative control, 9: molecular weight (100 bp).

The presence of the *icaA *(103 bp), *icaC *(400 bp) and *icaD *(198 bp) genes in the CNS isolates was demonstrated by the amplification of the corresponding fragments (Figure 1). The *icaA *and *icaD *genes were detected concomitantly in 40 (40%) of the 100 CNS isolates, and the *icaA*, *icaC *and *icaD *genes were detected concomitantly in 42 (42%). None of the genes studied could be identified in 18 (18%) isolates. Except for *S. haemolyticus *and *S. capitis*, all other CNS species were positive for the genes studied.

### Study of biofilm production by the tube adherence test (Christensen *et al. *1982)

Investigation of biofilm production by the tube adherence test showed that 82 of the 100 isolates were biofilm producers, including 44 strains isolated from catheter tips, 23 isolated by blood culture, and 15 isolated from nares specimens. With respect to species, 70 (85.4%) *S. epidermidis *isolates were positive. Biofilm production was also observed in *S. warneri *(n = 4), *S. cohnii *(n = 3), *S. xylosus *(n = 2), *S. saprophyticus *(n = 2), and *S. lugdunensis *(n = 1).

The sensitivity and specificity of the tube adherence test were calculated using the presence of the *ica *genes as a parameter. The results showed 100% sensitivity and 100% specificity of the tube test [24] when compared to PCR (concomitant presence of the *icaA *and *icaD *or *icaACD *genes).

### Study of biofilm production by the tissue culture plate method (Christensen *et al. *1985)

Among the 100 isolates studied, 35 (35%) were classified as weakly adherent and 46 (46%) as strongly adherent, for a total of 81 (81%) positive isolates and 19 (19%) negative isolates. Forty-four (54.3%) of the 81 positive isolates were obtained from catheter tips, 21 (25.9%) from blood culture, and 16 (19.8%) from nares specimens. Among the 19 negative isolates, 6 (31.6%) were obtained from catheter tips, 9 (47.4%) from blood culture, and 4 (21.0%) from nares specimens.

The TCP assay using a 540-nm filter presented 97.6% sensitivity and 94.4% specificity when compared to PCR recognizing the concomitant presence of the *icaA *and *icaD *or *icaACD *genes. One nares specimen tested false-positive, i.e., it was classified as strongly adherent but did not carry any of the *ica *genes. Two blood culture samples tested false-negative, i.e., they were classified as non-adherent but tested positive for the *icaACD *genes.

Analysis of agreement showed moderate agreement (kappa = 0.44) between the TCP assay and the concomitant presence of the *icaA *and *icaD *or *icaACD *genes.

To determine the relationship between the amount of biofilm and the concomitant presence of the *icaACD *genes, the positive results classified as strongly adherent obtained by spectrophotometric analysis of dry plates using a 540-nm filter were compared to those obtained by PCR. The results showed that 26 (56.5%) of the 46 strongly adherent isolates carried the *icaACD *genes. Moderate agreement (kappa = 0.44) was observed between strongly adherent isolates and the concomitant presence of the *icaACD *genes.

### Study of biofilm production by the Congo red agar method proposed by Arciola *et al. *(2001)

The international reference strains *S. simulans *ATCC 27851 and *S. xylosus *ATCC 29979, used as positive control, exhibited a bright black color, whereas *S. epidermidis *ATCC 12228 and *S. warneri *ATCC 10209 (negative controls) formed bordeaux and bordeaux metallic colonies, respectively.

Among the 100 CNS isolates studied, 73 formed black colonies on CRA, including bright black colonies in 41 and dry black colonies in 32. The results revealed 27 non-biofilm producers whose colony color ranged from pink (2%) to red (7%) and bordeaux (18%). Two variants were observed among black colonies (BB), one with a bordeaux spot in the center and the other with a red spot. The center of these colonies was removed and subcultured for 24 h on CRA to obtain pure isolates. After isolation, the two subcolonies were found to be negative, presenting a red and bordeaux color, respectively. After reisolation from the center, these isolates also tested negative by the tube test and TCP assay and the *icaADC *genes were not detected by PCR.

Nine isolates formed bordeaux colonies and were therefore considered to be negative. However, these isolates tested positive for the concomitant presence of the *icaA *and *icaD *genes or *icaACD *genes and were classified as false-negative when compared to PCR. Two of these isolates were obtained by blood culture and seven were isolated from nares specimens.

The CRA method presented 89% sensitivity and 100% specificity when compared to PCR recognizing the concomitant presence of the *icaA *and *icaD *or *icaACD *genes.

### Comparison of biofilm detection methods in CNS

Table 2 shows the comparison between the phenotypic methods for the detection of biofilm production and the presence of the *icaA *and *icaD *genes or *icaACD *genes. The tube adherence test was the phenotypic method showing the best sensitivity (100%) and specificity (100%) in biofilm detection when compared to PCR.

**Table 2:** Sensitivity and specificity of phenotypic methods for the detection of biofilm production in coagulase-negative staphylococci

Method		*ica *Positive^*^	*ica *negative^**^	Sensitivity %	Specificity %
Tube test	Adherent	82	0	100	100
	Non-adherent	0	18		

Plate test	Adherent	80	1	97,6	94,4
	Non-adherent	2	17		

CRA test	Adherent	73	0	89	100
	Non-adherent	9	18		

* *ica *positive: Positive for *icaA *and *icaD *or *icaACD.*

** *ica *negative: Negative for *icaA *and *icaD *or *icaACD.*

CRA: Congo red agar.

## Discussion

In view of the large number of infections caused by biofilm-producing bacterial, a reliable method for their diagnosis is necessary. In the present study, 100 CNS strains isolated from clinical specimens of newborns and from the nares of healthy subjects were analyzed. The results revealed *S. epidermidis *as the most frequently isolated species in both clinical and nares specimens, corresponding to 81% of all strains isolated. Other CNS species were also identified, including *S. cohnii*, *S. saprophyticus*, *S. warneri*, *S. haemolyticus*, *S. xylosus*, *S. capitis*, and *S. lugdunensis*. Similar results have been reported in other studies [35,36]. The finding of *S. epidermidis *as the most frequently isolated species in most studies might be due to the fact that this microorganism possesses certain mechanisms that favor its adaptation to some sites, with this species being the most prevalent bacterium in human skin and mucosa [37].

The present results showed no difference in the frequency of biofilm production between isolates obtained from clinical samples and from nares specimens of healthy subjects, irrespective of the detection method used. Similar biofilm production by CNS strains isolated from different sources, including clinical specimens, environment and microbiota of healthy individuals, has also been reported by other investigators [35,38].

The *icaA *and *icaD *genes were detected concomitantly in 40% of the 100 CNS isolates studied and the *icaACD *genes in 42%. These results differ from those reported by Cafiso *et al. *[9] who also investigated the presence of genes involved in biofilm production. In that study, 35% of the isolates were positive for the *icaA *and *icaD *genes and only 4 isolates carried the *icaACD *genes. Some isolates only carried the *icaD *gene. In the present study, PCR was found to be an efficient method for detection of the *ica *operon. Other investigators also reported PCR to be an important tool for the identification of *ica *genes since the technique is simple, rapid and reliable and only requires minimal amounts of DNA [9,39]. PCR was used in this study as a reference for the phenotypic method based on several studies [9,39-41] that demonstrated the efficiency of this technique in detecting the genes of the *ica *operon. In addition, these genes are important virulence markers of clinical CNS isolates since their expression is associated with the production of PIA, the most clearly characterized component of staphylococcal biofilms.

With respect to the phenotypic methods, the tube adherence test presented 100% sensitivity and 100% specificity when compared to PCR recognizing the concomitant presence of the *icaA *and *icaD *or *icaACD *genes. Ruzicka *et al. *[40] also reported good sensitivity and specificity for the tube test and PCR when analyzing isolates obtained from infections. According to Morales *et al. *[10] and Cunha *et al. *[42], the test provides reliable results for biofilm detection in CNS and is adequate for routine use.

According to the present study and reports in the literature [43], the expression of the *ica *genes is highly variable and can be induced by variations in the culture conditions, such as an increase in the concentration of sugars or other substances that induce stress. Mathur *et al. *[44] also obtained better results when the glucose concentration of TSB was increased to 1% and the period of incubation was prolonged to 24 h. The addition of large amounts of sugar to a medium colonized with CNS induces a stress condition which, in turn, stimulates fermentation, thus increasing the production of PIA and consequent biofilm production [5].

In the present study, 81% of the isolates were positive in the TCP test, with the observation of moderate agreement (0.44) between this test and PCR recognizing the concomitant presence of the *icaA *and *icaD *or *icaACD *genes. Similar results have been reported by Arciola *et al. *[45] who used the TCP assay for the detection of biofilm-producing isolates, with 81.2% of the strains testing positive.

According to Gerke *et al. *[14], when the *icaC *gene is expressed concomitantly with the *icaA *and *icaD *genes, oligomers of up to 130 UDP-N-acetylglucosamine residues are synthesized and the production of PIA thus increases. To confirm the results reported by these authors and to determine the reliability of the TCP method in terms of the quantification of biofilm production, the relationship between strongly adherent isolates and the concomitant presence of the three *ica *genes (*icaA*, *icaC *and *icaD*) detected by PCR was evaluated. The results showed that 56.5% of strongly adherent isolates carried the three genes. Moderate agreement (0.44) was observed between strongly adherent isolates and the concomitant presence of the *icaACD *genes.

In the present study, the colonies grown on CRA presented diverse colors, thus, a five-color scale was adopted for comparison of the results of the CRA test and PCR in order to correlate the variation in colony color with the presence of the *ica *genes. Colonies presenting a bright black and dry black color were classified as positive and those presenting a red, pink or bordeaux color as negative. Color scales were also adopted in other studies using the CRA test for better diagnostic performance, but color tones different from those proposed in this study were used. Very black, black and almost black colonies were classified as positive and bordeaux, red and very red colonies as negative [31,46]. In the present study, two black color variants were observed, one with a bordeaux dot in the center and the other with a red dot. These colonies were removed and subcultured for 24 in CRA to obtain pure isolates. All isolated subcolonies were again plated onto CRA plates and were found to be negative, showing a bordeaux and red color, respectively, and no *icaADC *genes were detected by PCR, similar to the findings of Arciola *et al. *[46].

In the present study, 73% of the 100 isolates tested by the CRA method were positive. Similar results have been reported in the study of Cafiso *et al. *[9], in which 83% of CNS strains isolated from catheter-associated infections were positive in the CRA test. However, lower positivity rates in this test have been reported in other studies. In investigations on the detection of slime production by *S. epidermidis *strains isolated from catheter [46] and prosthesis-associated [31] infections, 49% and 57% positive isolates were identified by the CRA method, respectively. Using the CRA method, Silva *et al. *[36] observed biofilm production in only 25% of CNS strains isolated from clinical specimens of newborns in a neonatal intensive care unit.

Comparison between the CRA test and the results obtained by PCR revealed nine CNS isolates forming bordeaux colonies which tested positive for the concomitant presence of the *icaA *and *icaD *genes or *icaACD *genes, with these results thus being false-negative when compared to PCR. Arciola *et al. *[31] identified eight isolates whose colony color ranged from red to bordeaux and that were classified as negative; however, the *ica *genes were detected in all of them. Cafiso *et al. *[9] observed three CNS isolates that were negative for biofilm production in the CRA test but contained the complete *ica *operon. In another study, six isolates that carried the *icaA *and *icaD *genes detected by PCR were negative in the CRA test [46]. Although PCR detects the presence of genes irrespective of their expression, PCR-positive isolates should be considered to be potential biofilm producers.

The CRA test is easier and faster to perform than other phenotypic tests. However, it is slightly imprecise in the identification of positive isolates when compared to molecular analysis of the genes involved in biofilm production, a fact also observed by Fitzpatrick *et al. *[47].

The present results demonstrated the presence of intercellular adhesion genes (*icaACD*) and consequent biofilm production in most CNS isolates. The proportion of biofilm-positive/*ica*-positive versus biofilm-positive/*ica*-negative strains was 82:0%, 80:1% and 73:0% by the tube test, TCP assay and CRA, respectively. In the study of Jiang *et al. *[17], the proportion of biofilm-positive/*ica*-positive versus biofilm-positive/*ica*-negative strains was 22:2%. In another study investigating *S. epidermidis *clinical strains isolated from blood cultures, this proportion was 85:2% [48]. Qin *et al. *[49] studied the two biofilm-positive/*ica*-negative strains SE1 and SE4 identified among 101 clinical isolates of *S. epidermidis *collected at Ruijin Hospital, Shanghai, China [17]. The authors investigated the *aap *and *bhp *genes that induce an alternative PIA-independent mechanism of biofilm formation. However, the two genes were not detected in the SE1 and SE4 strains, but were present in the RP62A reference strain (biofilm-positive/*ica*-positive). These authors suggested a novel molecular mechanism mediating biofilm formation in SE1 and SE4 clinical isolates. This mechanism needs to be further investigated.

In the present study, the tube adherence test showed the best correlation with the PCR results and can be indicated for the routine detection of biofilm production in CNS.

## Conclusion

The present results demonstrated the presence of intercellular adhesion genes (*icaACD*) and consequent biofilm production in most CNS isolates. The presence of this biofilm, in turn, facilitates the development of infections by compromising the immune system of the patient and contributing to the failure of antibiotic therapy, which may result in recurrent infections and the emergence of multiresistant pathogens. In addition, the results suggest the use of the tube adherence test for the routine detection of biofilm production in CNS because of its easy application and low cost and because it guarantees reliable results with excellent sensitivity and specificity.

## Competing interests

The authors declare that they have no competing interests.

## Authors' contributions

AO: Responsible for conceiving the idea of the study, performed the microbiological tests, and wrote the article. MLRSC: Responsible for conceiving the idea, coordinating laboratory work, data analysis, and manuscript writing. All authors read and approved the final manuscript.
